# Natural language processing application to identify covert administration of medicines: development and pilot audit

**DOI:** 10.1192/bjb.2025.28

**Published:** 2026-04

**Authors:** Ninoslav Majkic, Jyoti Sanyal, Robert Stewart, Nicola Funnell, Delia Bishara

**Affiliations:** 1 South London and Maudsley NHS Foundation Trust, London, UK; 2 Centre for Translational Informatics, Institute of Psychiatry, Psychology & Neuroscience, King’s College London, London, UK; 3 Mental Health for Older Adults and Dementia Clinical Academic Group, South London and Maudsley NHS Foundation Trust, London, UK; 4 Institute of Psychiatry, Psychology & Neuroscience, King’s College London, London, UK; 5 Department of Psychology, Institute of Psychiatry, Psychology & Neuroscience, King’s College London, London, UK

**Keywords:** Consent and capacity, natural language processing, health informatics, old age psychiatry, dementias/neurodegenerative diseases

## Abstract

**Aims and method:**

The covert administration of medicines is associated with multiple legal and ethical issues. We aimed to develop a natural language processing (NLP) methodology to identify instances of covert administration from electronic mental health records. We used this NLP method to pilot an audit of the use of covert administration.

**Results:**

We developed a method that was able to identify covert administration through free-text searching with a precision of 72%. Pilot audit results showed that 95% of patients receiving covert administration (*n* = 41/43) had evidence of a completed mental capacity assessment and best interests meeting. Pharmacy was contacted for information about administration for 77% of patients.

**Clinical implications:**

We demonstrate a simple, readily deployable NLP method that has potential wider applicability to other areas. This method also has potential to be applied via real-time health record processing to prompt and facilitate active monitoring of covert administration of medicines.

The covert administration of medicine is the practice of concealing medications in food or drink to administer them without the patient’s knowledge. There are multiple legal and ethical issues associated with covert administration of medicines, primarily concerning the infringement of human rights.^1^ However, when patients are not consenting to treatment and lack capacity to make decisions about their care, covert administration is sometimes deemed justifiable under best interests principles and following appropriate discussions and consensus to ensure that patients receive appropriate care.

In England specifically, the Care Quality Commission provides guidance on how to approach administering medicines covertly if deemed necessary.^2^ This includes completing a mental capacity assessment, carrying out a statutory best interests meeting and seeking pharmacy advice on how to safely conceal the medication in foodstuff. However, an international literature review concluded that the practice of covert administration is common in older adults, and the decision to administer covert medications was surprisingly made by a single staff member in many instances.^3^

Quality improvement through auditing is essential to ensuring that standards of healthcare are met consistently. One of the barriers to auditing patient records is the large bodies of text that auditors must screen to identify information relevant to their audit, if this information is not in pre-structured fields. Natural language processing (NLP) is a computational linguistics method used to extract information from large bodies of text.^4^ The application of NLP methods to healthcare can lead to rapid and accurate extraction of important health data to facilitate care.^5^

We sought to develop NLP to identify instances where patients were recorded as receiving covert administration of medicines. We implemented this approach to complete an audit of the use of covert administration within a large mental healthcare provider.

## Method

This project was completed within the South London and Maudsley National Health Service (NHS) Foundation Trust (SLaM). SLaM is one of Europe’s largest healthcare providers for mental health and dementia and serves a population of 1.36 million residents in four south London boroughs. The Clinical Record Interactive Search (CRIS) database provides regulated access to de-identified information extracted from SLaM patient records, to be used by authorised researchers within a robust and patient-led data security model.^6^ We used the CRIS database to develop and evaluate the NLP application. CRIS has received ethical approval as an anonymised data resource (Oxford Research Ethics Committee C, reference 23/SC/0257) and a patient-led Oversight Committee reviews and approves all projects using the data platform, including this one. In relation to both the NLP development and manual review of longer text fields for the proof-of-concept audit described here, fully de-identified records were used with identifiers obscured in text, as well as removed from structured data. Patient consent was not required for this work.

To develop this application, we began by considering text around the keyword ‘covert’. Following this we used the CRIS database to search through sources of text entries (including correspondence, ward progress notes and assessments) and randomly selected 200 entries that included this ‘covert’ keyword. We screened these entries for specific patterns and associated keywords that could be used to ascertain required entities (i.e. actual use of covert administration) more robustly. It was ascertained that the following associated keywords were very commonly found close to the ‘covert’ primary search term: accept*, given, took, taken, administer* and medication. After identifying this pattern, we developed the NLP using the Java Annotation Patterns Engine (JAPE) in the General Architecture for Text Engineering (GATE) software on Microsoft Azure.^7^

The rules created were as follows:

- compulsory presence of the keyword ‘covert*’
- compulsory presence of the ‘medication’ keyword within 0–5 words of ‘covert’
- at least one of the other keywords implying administration within 0–5 words of ‘covert’.

Examples of correctly targeted text included the following: ‘covert medication administration’; ‘covert medication given’; ‘given covert medication’; ‘took the majority of her covert medication’.

To test the performance of the application, we measured precision (equivalent to positive predictive value): the proportion of relevant instances among those retrieved by the application.^8^ This was estimated by manual review of the full content of the associated text field (e.g. a given case note or letter), then further extended to other text entries for the same patient in order to evaluate and confirm whether the patient had indeed received covert medication administration.

To evaluate the applicability of the NLP algorithm, we conducted an audit of the use of covert administrations identified. The standards were based on the Care Quality Commission guidance on covert administration of medicines and the trust’s own medicines policy. The audit criteria and standards are outlined in Table 1. A bespoke covert medication administration form in the trust requires all other aspects of the audit criteria to be completed and is contained in the electronic health record, serving as a prompt for clinicians to meet the audit standards described. To gather our data, we first investigated whether the covert administration form was completed. If this was completed, we could confirm that all three other audit standard criteria were met and were considered completed. If this was not present, records were manually inspected using CRIS to identify whether the other three audit criteria were met. Results for the audit criteria were then stratified by Clinical Academic Group (CAG); CAGs are locally defined groups within SLaM that contribute to research and quality improvement in clinical practice within their area.

**Table 1:** Audit criteria and standards for covert administration of medicines

Audit criterion	Standard
Mental capacity assessment	All patients who are administered medicines covertly must have a documented mental capacity assessment completed before first administration
Best interests meeting	For all patients, a best interests meeting must have taken place before administering medicines covertly
Pharmacy advice	For all patients, pharmacy advice should be sought before administering medicines covertly
Covert administration form	All patients who are administered medicines covertly must have a completed covert administration form on their record

## Results

We carried out precision testing of 100 individual instances of covert administration that were identified by the search. A result was deemed ‘positive’ if the text entry extracted by the application clearly demonstrated that the patient was receiving covert administration. Of the 100 instances, 72 (72%) were deemed a positive result. The application had extracted multiple entries of covert administration for some patients in our sample, and as a result 26 of the 100 instances were a subsequent record of covert administration for a patient already identified. When we evaluated the precision by including only one instance per patient, we found that 49 out of 74 instances (66%) were deemed a positive result.

For the 49 patients that had a recorded instance of covert administration, we explored the anonymised patient record around the date of the text entry. When scrutinising the patient records, we confirmed that 43 patients (88%) had received covert administration. For two patients (4%), covert administration was planned but not enacted; three patients (6%) had received covert administration in another healthcare service without input from SLaM services; one patient (2%) was identified where the family was administering medication covertly without any input or approval from healthcare professionals.

**Fig. 1:**
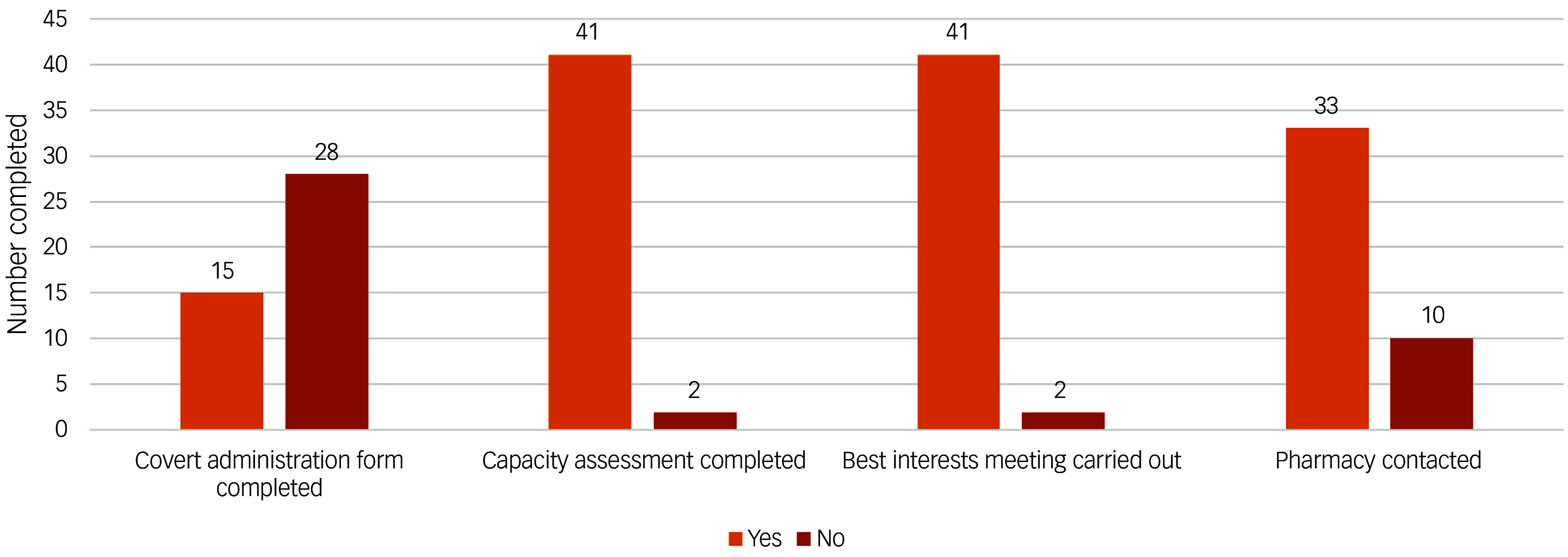
Audit results for all patients (*n* = 43) identified to have received covert medicines.

**Fig. 2:**
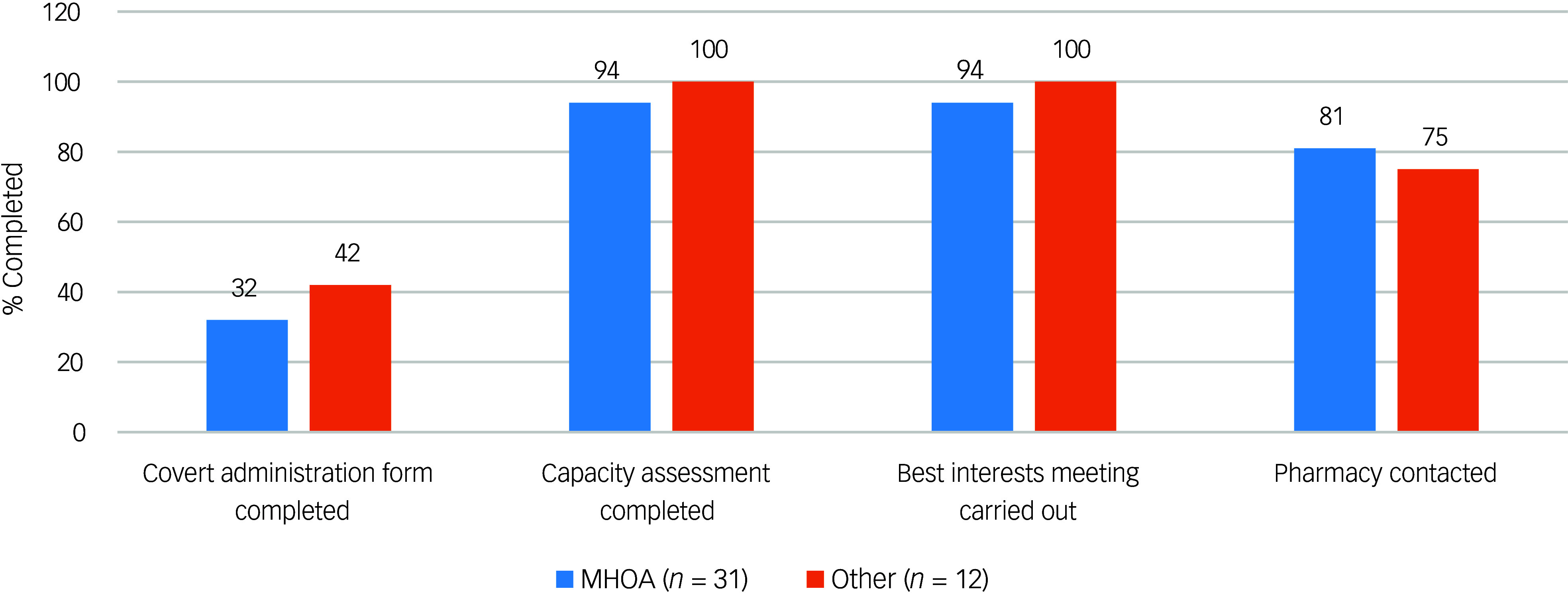
Audit results for patients identified to have received covert medicines (*n* = 43) stratified by the Clinical Academic Group (CAG) responsible for the patient’s care at the time of administration. ‘Other’ CAGs include Acute Care Pathway, Psychosis, Behavioural and Developmental Psychiatry, Children and Adolescent Mental Health Services, Neurodevelopmental, and patients where CAG was not documented. MHOA, Mental Health for Older Adults and Dementia CAG.

The results of the initial audit for the 43 patients included are outlined in Fig. 1. Most patients had received a capacity assessment and best interests meeting prior to initiation of covert medication. Fig. 2 displays the audit results stratified by CAG. The majority of covert administrations were carried out by the Mental Health for Older Adults and Dementia (MHOA) CAG.

## Discussion

Since the digitalisation of healthcare records, healthcare bodies have had quicker access to large amounts of important data relevant to service quality assessments. In many specialties such data will be in easily extractable (i.e. pre-structured) forms that can be contextualised to carry out quality improvement and research, such as blood investigation results and electronic prescription of medications. However, in mental healthcare the service quality data that are most important are often found within large text entries. Although one approach is to encourage the routine completion of structured forms in the record for particular purposes, this can be challenging to sustain without continued focus, and the level of completion of forms still requires monitoring against case note entries. There is therefore a need to develop tools that can extract relevant data from these large text entries to facilitate appropriate care evaluation.

We developed an NLP approach that was able to identify instances of covert administration of medication within text entries to an acceptable level (72% precision at instance level) and in a way that could be readily automated for routine output. Although higher precision could potentially be achieved with further adjustment of the search criteria, we anticipated that the number of instances of covert administration was low, so a higher precision threshold was not deemed necessary to pilot this tool, particularly as it was relatively straightforward to double-check identified instances manually.

We were able to use this tool to facilitate an audit of the use of covert administration of medicines. Our audit results showed that national standards were largely met. There were only 2 out of 43 instances where capacity assessments and best interests meetings were not documented before administration of covert medicines. However, we did find that there was a larger number of instances where pharmacy staff had not been contacted to confirm the suitability of administering medicines covertly (*n* = 10). Furthermore, most patients did not have a covert administration form completed. It appears therefore that although completion of the covert administration forms did not necessarily improve the rate of capacity assessments and best interests meetings being undertaken, it may act as a reminder for prescribers to seek pharmacist advice on how to safely administer medication covertly in foodstuff. This is important since not all tablets can be crushed (e.g. modified release preparations) and some medicines cannot be taken with certain foods or drinks (e.g. tetracycline antibiotics and iron supplements should not be taken with milk owing to reduced absorption). It is always important therefore to seek advice regarding this practice so as not to reduce the efficacy or alter the pharmacokinetics of medicines given in this way.

Similar studies utilising the CRIS database for novel quality improvement initiatives in mental healthcare have been published. One such study investigated the use of an antipsychotic initiation form to support compliance with standards for initiating antipsychotics in older adults with dementia, which found that real-time monitoring for safe prescribing was feasible in this way.^9^ Another study found benefits in the use of NLP to identify symptom presentation in text entries in people with first-episode psychosis.^10^ Electronic health records, still a relatively recent resource in many settings, provide novel opportunities for quality improvement in routine practice; however, this often depends on being able to ascertain required information at scale, much of which may be in text rather than structured fields.

### Strengths and limitations

A major strength of our use of NLP is its ability to extract data rapidly from patient records. Previously, we have relied on free-text searches of the patient record database for the single word ‘covert’ to produce an audit population. This led to a large number of false positives, which a clinician would have to review manually, resulting in a time-consuming process of reading and categorisation. The benefit of our application is that it accurately identifies covert administrations, with the potential to save clinician time in audit cycles and other contexts for ascertainment and evaluation of covert medication use. Running the application in our database took approximately 2–3 h. This approach also has potential to be applied in ‘real-time’, where an instance of covert administration is automatically flagged and brought to a service’s attention. This could allow for active monitoring of the use of covert administration, rather than relying on audits that happen at reduced intervals, potentially months or even years after patients first start receiving medicines covertly. Therefore, although the CRIS data platform was used to develop the NLP, its implementation is likely to be best configured as a routine run over source health records data, which should be readily accessible to digital services and/or business intelligence staff within healthcare provider organisations; this would maximise the potential for real-time processing, bypassing any limitation in data refresh frequencies adopted by different CRIS or equivalent platforms.

Our application was developed specifically for use within one mental healthcare provider (SLaM); however, it has potential to be applied to other databases in other trusts and healthcare settings, such as general hospitals. Our NLP technique is based on how clinicians record covert administration, and since it uses linguistic rules it may be more feasible to implement in other data sources than a measurement relying on structured forms, which are more likely to vary between services in their configuration and uptake. An advantage of using free-text methods is that it allows the application to be applied to several elements of a patient record. This further strengthens the application’s ability to be implemented in other healthcare systems. The rules used for the algorithm in this application were relatively simple and it should be eminently feasible for a database administrator to run the algorithm in our trust’s cloud-based system, and the structured query language (SQL) table output lends itself to visualisation for clinicians. As many other mental healthcare providers use similar cloud-based systems, the application’s simplicity has a clear benefit in that it can quite easily be applied in other settings where text searching is possible, with some adaptations made to address differences in recording between systems. Clearly, however, although we do not envisage substantial barriers to wider use, any future cross-service applicability would need further evaluation.

We were able to utilise the application to identify the covert administration form’s usage as a key area for improvement in clinical practice. Because the low numbers of covert administration forms completed might reflect a lack of awareness of the form, interventions in training and/or awareness-raising would be obvious next steps, followed by a repeat audit cycle. Our application may prompt quality improvement initiatives, such as the implementation of a bespoke covert administration module within our existing electronic prescribing system to improve recording.

The precision level of the approach is an area that could be improved with future work. As there are not many instances where medicines are administered covertly in SLaM alone, a lower threshold for precision was found to be generally acceptable. However, future improvements, such as utilising individual medication names instead of the term ‘medication’ as part of the search, may allow for a higher precision and sensitivity for the search. In addition, other search terms for ‘medication’ (e.g. ‘med*’, ‘medicine’, ‘drug’) might have improved precision. Recall (sensitivity) testing was planned but it was not feasible to carry this out for the application. Aside from the low number of covert administration events, the methods that would have to be used to identify a population with known covert medication use for recall testing would be very similar to the application’s search rules, which would have led to bias. Finally, the applicability of the approach and algorithm we have developed is predicated on covert medication being recorded in the first place, since it would not be able to pick up unrecorded instances. However, it is hoped that an enabled audit cycle allows attention to be drawn to covert medication use and its recording to be improved as a result – this again should be feasible to monitor empirically.

### Implications

This study has shown that a relatively simple, readily deployable NLP technique can be applied to electronic health records to evaluate whether standards for the use of covert administration of medicines have been met. This application has utility as a time-saving and cost-effective method to audit the use of covert medicines and to ensure high quality of care. The application could also potentially be utilised for real-time monitoring of covert administration of medicines.

## Data Availability

The data included in this article, together with additional data, are available from the corresponding author, N.M., on reasonable request.

## About the authors

**Ninoslav Majkic** is a pharmacist with South London and Maudsley NHS Foundation Trust, London, UK. **Jyoti Sanyal** is Natural Language Processing Lead in the Centre for Translational Informatics, Institute of Psychiatry, Psychology & Neuroscience, King’s College London, London, UK. **Robert Stewart** is Professor of Psychiatric Epidemiology and Clinical Informatics at the Institute of Psychiatry, Psychology & Neuroscience, King’s College London, London, UK, and a member of the Mental Health for Older Adults and Dementia Clinical Academic Group at South London and Maudsley NHS Foundation Trust, London, UK. **Nicola Funnell** is Clinical Director of Mental Health Services for Older Adults at South London and Maudsley NHS Foundation Trust, London, UK, and a member of the Mental Health for Older Adults and Dementia Clinical Academic Group, South London and Maudsley NHS Foundation Trust, London, UK. **Delia Bishara** is a consultant pharmacist with the Mental Health for Older Adults and Dementia Clinical Academic Group at South London and Maudsley NHS Foundation Trust, London, UK, and a researcher in the Department of Psychology at the Institute of Psychiatry, Psychology & Neuroscience, King’s College London, London, UK.
